# Primary admission to a surgical service facilitates early cholecystectomy in acute cholecystitis but does not influence patient outcome

**DOI:** 10.1007/s00423-023-02957-7

**Published:** 2023-06-05

**Authors:** Jens Strohäker, Julia Sabrow, Anke Meier, Alfred Königsrainer, Ruth Ladurner, Can Yurttas

**Affiliations:** Department of General, Visceral and Transplantation Surgery, University Hospital of Tuebingen, Hoppe-Seyler-Straße 3, 72076 Tuebingen, Germany

**Keywords:** Acute calculous cholecystitis, Admission policies, perioperative care, patient outcome, antibiotic stewardship

## Abstract

**Purpose:**

Early cholecystectomy is recommended for acute calculous cholecystitis to reduce complications and lower health care costs. However, not all patients admitted to emergency services due to acute calculous cholecystitis are considered for surgery immediately. Our intention was therefore to evaluate patient management and outcome parameters following cholecystectomy depending on the type of emergency service patients are primarily admitted to.

**Methods:**

We performed a retrospective analysis of all patients that were treated for acute cholecystitis at our hospital between 2014 and 2021. Only patients that underwent surgical treatment for acute calculous cholecystitis were included. Patients with cholecystectomies that were performed due to other medical conditions were not incorporated. Primary outcomes were the perioperative length of stay and postoperative complications. Perioperative antimicrobial management and disease deterioration according to Tokyo Guidelines from 2018 due to inhouse organization were assessed as secondary outcome parameters.

**Results:**

Of 512 patients included in our final analysis, 334 patients were primarily admitted to a surgical emergency service (SAG) whereas 178 were initially treated in a medical service (MAG). The latency between admission and cholecystectomy was significantly prolonged in the MAG with a median time to surgery of 2 days (Q25 1, Q75 3.25, IQR 2.25) compared to the SAG with a median time to surgery of 1 day (Q25 1, Q75 2, IQR 1) (*p* < 0.001). The duration of surgery was comparable between both groups. Necrotizing cholecystitis (27.2% vs. 38.8%, *p* = 0.007) and pericholecystic abscess or gallbladder perforation (7.5% vs. 14.6% *p* = 0.010) were less frequently described in the SAG. In the SAG, 85.7% of CCEs were performed laparoscopically, 6.0% were converted to open, and 10.4% were performed as open surgery upfront. In the MAG, 80.9% were completed laparoscopically, while 7.2% were converted and 11.2% were performed via primary laparotomy (*p* = 0.743). Histologically gangrenous cholecystitis was confirmed in 38.0% of the specimen in the SAG compared to 47.8% in the MAG (*p* = 0.033). While the prolonged preoperative stay led to prolonged overall length of stay, the postoperative length of stay was similar at a median of 3 days in both groups.

**Conclusions:**

To our knowledge, we present the largest single center cohort of acute calculous cholecystitis evaluating the perioperative management and outcome of patients admitted to either medical or surgical service prior to undergoing cholecystectomy. In patients that were primarily admitted to medical emergency services, we found disproportionately more gallbladder necrosis, perforation, and gangrene. Despite prolonged time intervals between admission and cholecystectomy in the MAG and advanced cases of cholecystitis, we did not record a prolonged procedure duration, conversion to open surgery, or complication rate. However, patients with acute calculous cholecystitis should either be primarily admitted to a surgical emergency service or at least a surgeon should be consulted at the time of diagnosis in order to avoid disease progression and unnecessary health care costs.

## Introduction

Symptomatic gallstones and acute calculous cholecystitis (ACC) are among the most commonly treated diseases in emergency medicine and surgery departments. In Germany in 2019, 203,563 cholecystectomies (CCE) were performed [1] of which around a third were done for ACC [2]. Since the ACDC-Trial in 2013 most German surgical departments have adopted an early CCE strategy [3]. Early CCE (within 72 h of symptom onset) has been shown to reduce complications and lower costs and is thus recommended by several guidelines [4–8]. Patients unfit for surgery are generally treated medically with anti-inflammatory drugs and antibiotics, sometimes accompanied by interventional drainage of the gallbladder [7]. The most recent version of the Tokyo Guidelines from 2018 (TG 2018) offers comprehensive severity grading and perioperative management recommendations [9].

While ubiquitous in many countries around the globe, Germany only recently made it compulsory to establish interdisciplinary emergency rooms and thus has seen an increase in interdisciplinary emergency contacts [10]. Given that there is no formal emergency medicine specialty, upper abdominal pain is frequently seen by either internal medicine or surgery personnel. Depending on the inhouse organization, patients with ACC are either admitted to the medical or surgical service. Medical admission, however, has been shown to decrease the rate of (early) cholecystectomy [11].

The goal of our study was to evaluate whether the perioperative patient management and postoperative outcome of patients admitted for ACC was different based on what service the patients were admitted to. Primary outcomes were the perioperative length of stay and complications. The secondary outcomes were perioperative antimicrobial management and patient outcome subject to inhouse organizational delays leading to an upgrade in disease severity.

## Material and methods

This is a retrospective analysis of all consecutive adult patients (i.e., > 18 years) that underwent cholecystectomy for acute calculous cholecystitis at our hospital. The database was generated searching for all patients that had undergone cholecystectomy between January of 2014 and January of 2021. All procedures in Germany are encoded using the “Operationen and Prozeduren Schluessel” (OPS-Code), and thus, we selected all patients that held the OPS-Codes 5-511.0, 5-511.1, and 5-511.2. (open, laparoscopic, and laparoscopic converted to open cholecystectomy as an independent procedure). Patients that underwent simultaneous cholecystectomy during a different procedure were excluded. In the next step, we excluded all patients that were hospitalized in a service different than the medical or surgical one at the time of cholecystitis. In the last step, we excluded all patients that were taken to the OR for acute calculous cholecystitis which was ultimately ruled out by the histology report (chronic cholecystitis, tumor).

### Inhouse organization

Our tertiary teaching hospital is the sole provider for all forms of gallstone disease in our district. During the study period, the hospital ran medical and surgical emergency room (ER) that were located in different sections of the same building complex. Patients were either referred to the medical or surgical ER at the discretion of the primary care physician or the paramedic first encountering the patient. Depending on the primary exam results and suspected diagnosis, surgery was involved sooner or later during the course of disease. When a patient was diagnosed with ACC admission to either the medical or surgical ward was done, based on different factors such as availability of a bed, projected OR capacities and/or comorbidities. Both the general surgery department and the department of gastroenterology share an endoscopy unit. Endoscopic retrograde cholangiography is provided by a total of five different attendings (two surgical and three medical endoscopists) indifferent of what service the patient had been admitted to. After the CCE patients, who had not yet been admitted to surgery were transferred to a surgical ward in order to facilitate post-op visitations, follow-up and discharge.

### Surgery for acute calculous cholecystitis

All cholecystectomies were performed under general anesthesia. If patients were on empiric antibiotic therapy for cholecystitis, no additional prophylactic single shot antibiotic was administered prior to skin incision. Laparoscopic cholecystectomy was performed in a standardized 4-port technique in supine German/American position, utilizing a 12 mm subumbilical port, a 10-mm subxyphoidal port and two 5-mm ports placed in the right subcostal area. Access to the peritoneal cavity and insufflation of CO_2_ was established by a Veress-needle. If laparoscopic cholecystectomy was considered feasible, the triangle of Calot was first dissected for definite identification of the cystic duct and artery in a critical view of safety [12] before transection and releasing the remaining gall bladder from its attachments to the liver in a retrograde approach. In case of conversion to open cholecystectomy or primary open cholecystectomy median laparotomy was the preferred approach over subcostal or L-shaped horizontally extended incisions. A surgical drain was placed at the surgeon’s discretion. No routine cholangiography was performed.

### Statistics

All comparisons between groups were carried out by Chi-square test or Fisher’s exact test for nominal variables and Mann–Whitney *U* test or Kruskal–Wallis test for continuous variables, whichever was applicable. A probability of less than 0.05 was considered to be statistically significant. All *p* values reported are results of two-sided testing. Where needed, Bonferroni-correction was applied. Statistical analysis was carried out using IBM Statistics for the Social Sciences (SPSS) for Windows, Version 28.0 (IBM Corp., Armonk, NY, USA).

## Results

### Patient cohort

A total of 512 patients were included in the final analysis. Around two-thirds of the patients were admitted to the surgical service. The median age was 66 years and was comparable between both the medical and surgical group. Of all 512 patients admitted to the medical and surgical department, 39.9 % and respectively 47.6 % were women (*p* = 0.073). In the medical admission group (MAG), the ASA-Scores were higher than in the surgical admission group (SAG). This was due to a higher number of ASA III patients at the expense of less ASA I & II patients. Both groups had 3.3% of ASA IV patients. Both cohorts were comparable with regards to their BMI (*p* = 0.606) and weekday of admission (*p* = 0.526). The use of oral anticoagulation (both direct thrombin inhibitors and vitamin k antagonists) was similarly distributed between patients of both cohorts (11.7% SAG vs. 14.0% MAG). With regards to external referrals 81.1% of the patients in the SAG and 75.3% of the MAG were from the hospital’s district. Overall, 12.6% and 18.0% were from neighboring districts while 6.2% and 6.7% of patients were from distant districts (*p* = 0.236). For details, see Table 1.

**Table 1:** Patient outcome according to admission

	Overall cohort *n* = 512	Medicine (MAG) *n* = 178 (34.8%)	Surgery (SAG) *N* = 334 (65.2%)	P*
Age in years, median (IQR)	66 (Q25 54, Q75 78, IQR 24)	67 (Q25 55, Q75 76, IQR 21)	65 (Q25 52, Q75 79, IQR 27)	0.911
Elderly patients (≥ 60 years)	317 (61.9%)	112 (62.9%)	205 (61.3%)	0.732
Sex, female (%)	229 (44.7%)	76 (39.3%)	159 (47.6%)	0.073
ASA-score				0.031
- I	58	13 (7.3%)	45 (13.5%)	
- II	262	85 (47.8%)	177 (53.0%)	
- III	175	74 (41.6%)	101 (32.2%)	
- IV	17	6 (3.3%)	11 (3.3%)	
- V	0	0	0	
Coronary artery disease	92 (18.0%)	41 (23.0%)	51 (15.3%)	0.029
Oral anticoagulants	64 (12.5%)	25 (14.0%)	39 (11.7%)	0.440
Admission imaging				
- Ultrasound	430 (84.0%)	145 (81.5%)	285 (85.4%)	0.256
- CT-scan	272 (53.1%)	114 (64.0%)	158 (47.3%)	<0.001
Pre-operative ERC	88 (17.2%)	32 (18.0%)	56 (16.7%)	0.729
Latency between admission and CCE in days, median (IQR)	1 (Q25 1, Q75 2, IQR 1)	2 (Q25 1, Q75 3.25, IQR 2.25)	1 (Q25 1, Q75 2, IQR 1)	< 0.001
Total length of stay in days, median (IQR)	5 (Q25 4, Q75 8, IQR 4)	6 (Q25 4, Q75 10, IQR 6)	5 (Q25 3, Q75 8, IQR 5)	< 0.001
Postoperative length of stay in days, median (IQR)	3 (Q25 2, Q75 6, IQR 4)	3 (Q25 3, Q75 6, IQR 3)	3 (Q25 2, Q75 6, IQR 4)	0.118
Severity (TG 2018, on admission)				0.024
- Mild	272 (52.7%)	86 (48.3%)	186 (55.7%)	
- Moderate	203 (39.6%)	71 (39.9%)	130 (38.9%)	
- Severe	39 (7.6%)	21 (11.8%)	18 (5.4%)	
Severity (TG 2018, during surgery)				0.004
- Mild	134 (26.2%)	37 (20.8%)	97 (29.0%)	
- Moderate	337 (65.8%)	118 (66.3%)	219 (65.6%)	
- Severe	41 (8.0%)	23 (12.9%)	18 (5.4%)	
Intraoperative findings				
- Necrotizing cholecystitis	160 (31.2%)	69 (38.8%)	91 (27.2%)	0.007
- Perforation/abscess	51 (10.0%)	26 (14.6%)	25 (7.5%)	0.010
Complicated cholecystitis	224 (43.8%)	94 (53.8%)	130 (38.9%)	0.003
Histologic results				
- Gangrenous cholecystitis	212 (41.4%)	85 (47.8%)	127 (38.0%)	0.033
Duration of antibiotic treatment in days, median (IQR)				
- Pre-operative	2 (Q25 0, Q75 3, IQR3)	2 (Q25 0, Q75 3, IQR 3)	1 (Q25 0, Q75 3, IQR 3)	0.006
- Post-operative	3 (Q25 2, Q75 5, IQR3)	3 (Q25 2, Q75 5, IQR 3)	3 (Q25 2, Q75 5, IQR 3)	0.596

### Preoperative management

At admission more than 80% of patients received an abdominal ultrasound in both groups (*p* = 0.256). The number of patients that had an (additional) abdominal CT-scan was significantly higher in the MAG with 64.0% vs. 47.3% (*p* < 0.001). The need for — and performance of — a preoperative endoscopic retrograde cholangiography (ERC) was independent of the admission to either service (18.0% vs. 16.7%, *p* = 0.729). The duration of symptoms until admission was documented for 439 patients (85.7%). Patients in both groups presented after a median duration of 1 day of abdominal discomfort/pain. The latency between admission and cholecystectomy was significantly prolonged in the MAG with a median time to surgery of 2 days (Q25 1, Q75 3.25, IQR 2.25) compared to the SAG with a median time to surgery of 1 day (Q25 1, Q75 2, IQR 1) (*p* < 0.001).

### Intraoperative findings

The duration of the procedure was comparable between both groups. The median duration was 95 minutes for the patients in the SAG (mean 101 min, STD 41, range 28 to 329 min, Q25 74, Q75 122, IQR 48) compared to 93 min for the patients admitted to medicine (mean 97 min, STD 37, range 30–218, Q25 71, Q75 116, IQR 45) (*p* = 0.302). Necrotizing cholecystitis was less frequently described in the SAG (27.2% vs. 38.8%, *p* = 0.007). Pericholecystic abscess or gallbladder perforation was also less frequent in the SAG (7.5% vs. 14.6% *p* = 0.010). In the SAG, 85.7% of CCEs were performed laparoscopically, 6.0% were converted to open and 10.4% were performed as open surgery upfront. In the MAG 80.9% were completed laparoscopically, while 7.2% were converted and 11.2% were performed via primary laparotomy (*p* = 0.743). The most common reason for primary open CCE was severe cardiovascular comorbidity without anesthesia clearance for laparoscopy (*n* = 14, 25%) followed by extensive inflammation on imaging with adjacent liver abscesses and/or sepcticemia from free perforation (*n* = 10, 18%) and recent history of surgery/laparotomy (*n* = 8, 14%). The three leading causes for conversion to open were unidentifiable structures in Calot’s triangle and inflammation that made laparoscopic dissection perilous (*n* = 15, 45%), substantial adhesions from previous procedures (*n* = 6, 18%), and intraoperative bleeding/ vascular injury (*n* = 5, 15%).

### Postoperative outcome and management

Histologically gangrenous cholecystitis was confirmed in 38.0% of the specimen in the SAG compared to 47.8% in the MAG (*p* = 0.033). The percentage of acutely exacerbated chronic cholecystitis was higher in the SAG although that was not statistically significant (*p* = 0.081). Postoperative complications were detected in 11.9% of the SAG compared to 12.4% in the MAG (*p* = 0.899). While the prolonged preoperative stay led to prolonged overall length of stay, the postoperative length of stay was similar in both groups at a median of 3 days in both group with a slightly wider IQR in the surgical group and thus earlier dismissal of the lowest quartile (*p* = 0.118).

### Perioperative antibiotics

Overall 327 patients received preoperative antibiotics (63.9%). Preoperative antibiotics were administered in 61.1% of patients in the SAG compared to 69.7% of patients in the MAG. This difference was not statistically significant (*p* = 0.054). The median duration of preoperative antibiotics was 1 day in the SAG (Q25 0, Q75 3, IQR 3) compared to a median duration of 2 days in the MAG (Q25 0, Q75 3, IQR 3) (*p* = 0.006). A total of 329 patients had continued antibiotic treatment postoperatively. That accounted for 63.6% of the patients in the SAG and 66.9% of patients in the MAG (*p* = 0.371). The median duration of postoperative antibiotic therapy in both groups was identical at 3 days (Q25 2, Q75 5, IQR 3).

### Choice of antimicrobial substance

The preoperative antimicrobial selection was significantly different between the SAG and the MAG. In descending order, the surgical service prescribed ciprofloxacin in *n* = 130 patients (63.7%), piperacillin/tazobactam in *n* = 43 (21.1%), ceftriaxone in *n* = 19 (9.3%) and other substances in *n* = 12 (5.9%). The medical service prescribed ciprofloxacin in *n* = 48 patients (38.7%), ceftriaxone in *n* = 33 (26.6%) and piperacillin/tazobactam in *n* = 22 (17.7%) as well as other substances in *n* = 21 (16.9%) (*p* < 0.001).

### Outcomes based on disease severity

At the time of admission patients were classified according to the Tokyo Guidelines as mild (*n* = 272, 53.1%), moderate (*n* = 201, 39.3%), or severe (*n* = 39, 7.6%). For details, see Table 2. Between the admission and the CCE 95 patients (18.6%) were upgraded to moderate cholecystitis for a duration of symptoms > 72h. Another 41 patients (8.0%) were upgraded to moderate cholecystitis due to intraoperative diagnosis of necrotizing cholecystitis, abscess or gallbladder perforation. Four patients were upgraded for developing white blood count of >18.000/ μl. Two patients were upgraded to severe cholecystitis due to developing septicemia between admission and CCE (one mild, one moderate on admission). Histologically gangrenous cholecystitis was detected in 5.2% of patients classified as mild cholecystitis at the timing of surgery while 26.3% of gallbladders upgraded from a mild to a moderate cholecystitis simply due to the duration of symptoms had histologic gangrene. Gangrenous cholecystitis was also present in 29.0% of all cases of moderate cholecystitis whose only criteria fulfilled was the duration of symptoms. For details, see Table 3.

**Table 2:** Outcomes according to TG 2018 on admission

Disease severity on admission	Mild *n* = 272 (53.1%)	Moderate *n* = 201 (39.3%)	Severe *n* = 39 (7.6%)	P*
Age in years, median (IQR)	60 (Q25 48, Q75 72, IQR 24)	71 (Q25 57, Q75 81, IQR 24)	78 (Q25 71, Q75 84, IQR 13)	< 0.001
Sex, female	125 (46.0%)	91 (45.3%)	13 (33.3%)	0.327
Coronary artery disease	41 (15.1%)	32 (15.9%)	19 (48.7%)	< 0.001
Oral anticoagulants	27 (9.9%)	24 (11.9%)	13 (33.3%)	< 0.001
CCE after hour or on weekend/holiday	97 (35.7%)	88 (43.8%)	19 (48.7%)	0.102
Admission imaging				
- Ultrasound	248 (91.2%)	161 (80.1%)	21 (53.8%)	< 0.001
- CT-scan	96 (35.1%)	142 (70.6%)	34 (87.2%)	< 0.001
Laparoscopic	247 (90.1%)	154 (76.6%)	14 (35.9%)	< 0.001
Conversion to open	13 (4.7%)	17 (8.5%)	3 (7.7%)	
Primary open	12 (4.4%)	30 (14.9%)	22 (56.4%)	
Latency between admission and CCE in days, median (IQR)	1 (Q25 0, Q75 2, IQR 2)	1 (Q25 1, Q75 2, IQR 1)	2 (Q25 1, Q75 5, IQR 4)	0.018 #
Total length of stay in days, median (IQR)	4 (Q25 3, Q75 5, IQR 4)	6 (Q25 4, Q75 9, IQR 5)	14 (Q25 8, Q75 19, IQR 11)	< 0.001
Postoperative length of stay in days, median (IQR)	3 (Q25 2, Q75 4, IQR 2)	4 (Q25 3, Q75 6, IQR 3)	9 (Q25 5, Q75 14, IQR 9)	< 0.001
Duration of surgery in minutes days, median (IQR)	92 (Q25 68, Q75 118, IQR 50)	94.0 (Q25 77, Q75 122, IQR 45)	102 (Q25 831, Q75 128, IQR 445)	0.087
White blood count (adm)	12,028 (± 3960)	15,887 (± 6304)	15,550 (± 6810)	< 0.001 *
c-Reactive protein (adm)	6.9 (± 8.5)	14.4 (± 12.1)	18.4 (± 12.4)	< 0.001 *
Intraoperative findings				
- Necrotizing cholecystitis	51 (18.8%)	86 (42.8%)	23 (59.0%)	< 0.001
- Perforation/abscess	10 (3.7%)	31 (15.4%)	10 (25.6%)	< 0.001
Complicated cholecystitis	70 (25.7%)	123 (61.2%)	31 (79.5%)	< 0.001
- Gangrenous cholecystitis	67 (24.6%)	117 (58.2%)	28 (71.8%)	< 0.001
Postoperative antibiotic continuation	148 (54.4%)	143 (71.1%)	38 (97.4%)	< 0.001
Postoperative duration of antibiotic treatment in days, median (IQR)	2 (Q25 1, Q75 4, IQR 3)	4 (Q25 2, Q75 5, IQR 3)	5 (Q25 4, Q75 8, IQR 4)	< 0.001

#Mild vs. moderate 0.074, mild vs. severe 0.084, moderate vs. severe 1.000 after Bonferroni correction

*Pairwise comparison mild vs. moderate *p* < 0.001, mild vs. severe < 0.001, moderate vs. severe *p* > 0.05

**Table 3:** Outcomes according to TG 2018 during surgery

Disease severity on admission	Mild *n* = 131 (25.6%)	Moderate *n* = 340 (66.4%)	Severe *n* = 41 (8.0%)	P*
Age in years, median (IQR)	55 (Q25 43, Q75 67, IQR 24)	67 (Q25 56, Q75 79, IQR 23)	78 (Q25 71, Q75 84, IQR 13)	< 0.001
Sex, female	70 (53.4%)	146 (42.9%)	13 (31.7%)	0.026
Coronary artery disease	11 (8.4%)	61 (17.9%)	20 (48.8%)	< 0.001
Oral anticoagulants	8 (6.1%)	42 (12.4)	14 (34.1%)	< 0.001
CCE after hour or on weekend/holiday	57 (43.5%)	127 (37.4%)	20 (48.8%)	0.225
Admission imaging				
- Ultrasound	123 (93.9%)	285 (83.8%)	22 (53.7%)	< 0.001
- CT-scan	28 (29.0%)	198 (58.2%)	36 (87.8%)	< 0.001
Laparoscopic	124 (94.7%)	276 (81.2%)	23 (56.1%)	< 0.001
Conversion to open	0 (0.0%)	30 (8.8%)	3 (7.3%)	
Primary open	7 (5.2%)	34 (10.1%)	15 (36.6%)	
Latency between admission and CCE in days, median (IQR)	1 (Q25 0, Q75 2, IQR 2)	1 (Q25 1, Q75 3, IQR 2)	2 (Q25 1, Q75 5, IQR 4)	< 0.001^&^
Total length of stay in days, median (IQR)	4 (Q25 3, Q75 5, IQR 2)	6 (Q25 4, Q75 9, IQR 5)	14 (Q25 8, Q75 19, IQR 11)	< 0.001
Postoperative length of stay in days, median (IQR)	2 (Q25 2, Q75 3, IQR 1)	4 (Q25 3, Q75 6, IQR 3)	8 (Q25 6, Q75 14, IQR 8)	< 0.001
Duration of surgery in minutes days, median (IQR)	90 (Q25 65, Q75 114, IQR IQR 49)	95.0 (Q25 75, Q75 122, IQR 47)	100 (Q25 81, Q75 125, IQR 44)	0.036 #
White blood count (preop) ± SD	11455 (± 3611)	13171 (± 6071)	13753 (± 7306)	0.114
c-Reactive protein (preop) ± SD	7.2 (± 9.4)	13.9 (± 10.1)	17.2 (± 11.1)	< 0.001^&^
Intraoperative findings				
- Necrotizing cholecystitis	4 (3.1%)	132 (38.8%)	24 (59.0%)	< 0.001
- Perforation/abscess	1 (0.8%)	39 (11.5%)	11 (26.8%)	< 0.001
Complicated cholecystitis	7 (5.3%)	185 (54.4%)	32 (78.0%)	< 0.001
- Gangrenous cholecystitis	7 (5.3%)	176 (51.8%)	29 (70.7%)	< 0.001
Postoperative antibiotic continuation	69 (52.7%)	220 (64.7%)	40 (97.6%)	< 0.001
Postoperative duration of antibiotic treatment in days, median (IQR)	2 (Q25 1, Q75 3, IQR 2)	3 (Q25 2, Q75 4, IQR 2)	5 (Q25 3, Q75 8, IQR 5)	< 0.001

#Mild vs. moderate 0.065, mild vs. severe 0.127, moderate vs. severe 1.000 after Bonferroni correction

^&^Mild vs. moderate *p* < 0.001, mild vs. severe *p* < 0.001, moderate vs. severe *p* 0.292

The number of criteria for moderate cholecystitis fulfilled at the time of admission ranged between zero and three in our cohort. Even when patients fulfilled zero criteria they were ultimately diagnosed with complicated cholecystitis in 25.6%. These numbers increased to 58.0% when one criterion was met, 82.5% with two criteria and 91.7% for three criteria. No patient fulfilled all four criteria from the TG 2018.

### Conservative treatment of cholecystitis

We additionally screened the hospital records for all patients that were classified with the International Classification of Disease-Code K81.0 (acute cholecystitis) but did not have one of the aforementioned procedure codes for cholecystectomy. Of the 224 patients, 52 patients were excluded from the analysis for various reasons. Most commonly because they did not fulfill the criteria of acute cholecystitis according to the TG 2018 in hindsight. Sixteen of these 52 patients had histologic evidence of acute cholecystitis but underwent CCE during other procedures (liver resections, pancreatic resections or bowel resections due to ischemia). Twenty-six additional patients were excluded because they were admitted to a department other than medicine or general surgery when the cholecystitis developed. A total of 146 patients remained that were either admitted to the general surgery or medicine service during the study period. Of these, 40 patients suffered from stress cholecystitis, thirty of which were treated in either the surgical or medical intensive care unit. None of these patients underwent interventional or operative treatment for cholecystitis. Ultimately 106 patients fulfilled the criteria of acute cholecystitis. Twenty-three of these were admitted surgically, and 83 were admitted medically. Eighteen patients (15.6%) suffered from metastatic malignancies or locally irresectable gallbladders due to tumor infiltration. In 18 cases (15.6%), either the patients or their legal guardian denied consent for CCE. Fourteen cases (12.1%) were classified as acalculous cholecystitis at the time or were coincidental with additional (mostly infectious) pathologies that put off surgery. In 10 cases (8.6%), surgery was never consulted regarding CCE. For one patient (0.9%), no reason could be identified why the CCE was not performed. Excluding the aforementioned reasons CCE was withheld in 55 patients for the following documented reasons. The most common was a simultaneous cardiac event (acute NSTEMI *n* = 14, congestive heart failure *n* = 3) or the need to continue anticoagulation *n* = 2 or dual platelet inhibition *n* = 3. The second most common reason was a simultaneous hepato-pancreato-biliary pathology in 9 cases (acute pancreatitis *n* = 4, patients with ACC on waiting list expecting liver transplant *n* = 2, acute liver failure *n* = 1, septic cholangitis *n* = 1, cholestasis due to IPMN *n* = 1). Eight patients were deemed to have more emergent comorbidities than ACC (pleural empyema *n* = 1, acute lymphatic leukemia *n* = 1, severe aplastic anemia *n* = 1, cytopenia due to recent chemotherapy *n* = 1, new onset epilepsy *n* = 1, indwelling ventriculoperitoneal shunt *n* = 1, acute colitis with simultaneous pericardial effusion *n* = 1). In 11 cases, the surgeon declined emergent CCE due to prolonged time since symptom onset (5–14 days) in patients that responded well to antibiotic treatment. In four patients, no surgical consult was ordered without a documented reason why and in the remaining two cases CCE should have been performed according to guidelines but the surgical consultant declined to do it for unknown reasons.

## Discussion

The inflammation of the gallbladder caused by gallstone obstruction is called acute calculous cholecystitis. Current guidelines recommend the surgical removal of the gallbladder at the earliest possible time and whenever possible within 72 h of symptom onset. However, it is considered safe within the first week of hospital admission [3, 5, 6, 8]. Since our tertiary care university hospital is the sole provider for treatment of gallstone disease in our district (including both for ERC and CCE), we assume that there is no referral bias of severe cases of acute cholecystitis to our hospital. During the time of analysis, the hospital provided outpatient care in two separate emergency rooms (one medical and one surgical). There are different hypotheses why patients may have been referred to either department. While age and gender were similar, there were slightly higher ASA-Scores in the MAG, so comorbidities, risk-profile, and overall patient condition may have influenced primary referrals. There is only a limited amount of studies that compared the outcomes of patients admitted to different services due to ACC. Macedo et al. reported on a cohort of 329 patients of which two-thirds were admitted to the medical service. Their medically admitted patients were significantly older and suffered from more (mainly cardiovascular) comorbidities [13]. Lu et al. described a tendency towards medical admission for patients presenting to the ED at night independent of medical comorbidities [14]. This may be influenced by the surgeon’s preference not to perform CCEs during the night after a 2014 study by Phatak et al. showed an increased morbidity for night-time CCEs [15]. Both Macedo et al. and Lu et al. concluded that surgical admission was favorable given the significantly shortened length of stay, time to cholecystectomy, and decreased costs [13, 14]. While we do not know what ultimately influenced admission to either service in our cohort, we did see less differences between the SAG and MAG. The ASA scores were lower in the SAG and the MAG featured a small, yet significant difference in the severity grade on admission. Our data confirms that medical admission leads to a delay in CCE compared to a surgical admission. The reasons for that may be manifold. Increased diagnostic workup [14] has been shown to delay CCE for patients admitted to surgery and preoperative interventions (ERC) may also delay the procedure. We can confirm that patients in the MAG group had significantly more CT-scans; however, ERCs were performed at the same rate in both our groups and the outcome of patients in the MAG group that had delayed CCE was not significantly different to those of the SAG.

Gonzales-Munoz et al. published a cohort of patients in 2014 where they compared the outcomes of patients admitted to the surgical, medical, or another department. They concluded that surgical patients were 5 times more likely to receive CCE than patients admitted to non-surgical services. What’s surprising is the overall low rate of only 51.5% of patients undergoing CCE in their cohort. While they did not explicitly state it, admission to a non-surgical service or development of ACC in a non-surgical ward may lead to suboptimal management and potentially less favorable outcome [11]. In our study, 106 patients with calculous cholecystitis were treated non-operatively during the acute episode. While this number still makes up for 17.2% of all patients with ACC most decisions to postpone or deny surgery altogether could be followed in hindsight. Only four patients were admitted to the medical service that never had a surgical consult; thus, we cannot replicate the rate of CCE in medically admitted patients.

Septic patients (grade III severe) were also more likely to be admitted to medicine and underwent CCE after initiation of antibiotic therapy. The 2013 Tokyo guidelines initially recommended non-surgical management for patients graded as severe. Shortly after, Amirthalingam et al. showed that severe cholecystitis could also be managed to be saved with surgery [16]. Data on the timing of CCE in severe cholecystitis remains scarce, however. Stabilizing the patient medically, followed by early source control should be the preferred strategy nonetheless. A recent Japanese cohort of 35 patients showed that for severe cholecystitis, “early” CCE (≤ 7 days after admission) was safe and lead to less conversions and a shorter overall length of stay [17].

The Tokyo guidelines grade disease severity based on several factors: Grade I (mild) is defined as the absence of either or any combination of the following criteria: (A) > 18.000/μl white blood count, (B) palpable tender mass in the right upper quadrant, (C) a symptom duration of > 72 h, or (D) evidence of marked local inflammation [9].

After admission 94 patients had to be upgraded from mild to moderate for the duration of symptoms > 72 h in our cohort. Forty-one patients were upgraded to moderate for the intraoperative findings of complicated cholecystitis and four for the development of leukocytosis of > 18.000/μl. Of the patients initially classified as mild cholecystitis that remained within the criteria of mild 5.2% had histologic evidence of gangrenous cholecystitis. In 96 patients that were initially classified as mild but were upgraded to moderate solely based on symptom duration, gangrene was present in 26.3%. This number is similar to the patients that initially presented with moderate cholecystitis due to symptom duration. While the outcomes of our patients were overall favorable, operating mild cases beyond the 72 hour bar may lead to prolonged procedures and hospital stay. Tur-Martinez et al. recently published a cohort of patients with > 5 days of symptoms or > 5 days duration between admission for ACC and CCE and concluded that these patients were not at an increased risk for complications [18].

To our knowledge, we present the largest single center cohort of acute calculous cholecystitis evaluating the perioperative management and outcome of patients admitted to either medical or surgical service prior to undergoing cholecystectomy. Of the 512 patients analyzed in this study two-thirds were admitted to the surgical service and one-third to the medical. Overall the patient characteristics were comparable with a slightly higher ASA-Score and a higher percentage of septic patients (11.8% vs. 5.4%) in the MAG. Taking into account the small number of septic patients (*n* = 21) in the MAG, we found disproportionately more gallbladder necrosis, perforation, and gangrene. Whether this was due to a prolonged preoperative period in the MAG is unknown. Fortunately, these advanced cases did not lead to prolonged procedure duration, conversion to open surgery, or complications, which is comparable to Tur-Martinez et al. [18]. Nonetheless, we observed a substantial amount of conversion to open and primarily open CCEs in our cohort, not all warranted by patient intolerance to capnoperitoneum. With the recent development of advanced techniques (such as fluorescence-guided dissection of Calot’s triangle) and the more widely accepted bail-out strategies of subtotal cholecystectomy, the rate of laparoscopic completion even in complex ACC will to be increased [19, 20].

Even though the supposedly more severe cases of cholecystitis were admitted to medicine the broad-spectrum antibiotics (mainly piperacillin/tazobactam) were more frequently prescribed by surgery. The differences in prescription based on the treating department leaves room for antibiotic stewardship interventions. Currently, no data supports the use of antibiotics broader than 3rd generation cephalosporins in the absence of sepsis and/or known resistant bacteria from blood or bile cultures aside from known MRGN carrier status. Whether the broader coverage in SAG lead to slower disease progression is speculative and open for debate. We have since adapted our antibiotic recommendation towards 3rd generation cephalosporins in non-septic patients. We strongly believe that antimicrobial strategies should be standardized within a hospital complex/group or trust based on local resistance patterns. The recently published global clinical pathways for intraabdominal infections that serve as a joint recommendation of several societies specialized in the perioperative management of infections, are an excellent framework to build a local a standard operating procedure from [21].

### Strengths and limitations

Due to the retrospective origin of this study some information is simply not available. The hospital information system neither documents the time of surgical consultation nor the time of the answer. Thus, delays are hard to attribute to either department and may have different underlying etiologies.

## Conclusion

Prolonged intervals between diagnosis of cholecystitis and cholecystectomy did not lead to unfavorable outcomes but generated higher costs, longer length of stay and was accompanied by more advanced histologic findings in the MAG. Thus, we recommend admitting patients with ACC to the surgical service to facilitate early laparoscopic cholecystectomy, whenever possible. Independent of local organization, patients with suspected ACC warrant an early surgical consult and should be seen in the ER right away. While CCE can and should be safely performed even a week into symptoms, histologic progression of disease may lead to advanced cases and avoidable prolonged hospital stays and antibiotic therapy.

## Data Availability

All data and material can be provided on further request.
